# Marketing-AutoM3L: domain-aware automated machine learning for financial customer analytics

**DOI:** 10.3389/frai.2026.1726900

**Published:** 2026-01-27

**Authors:** Ye Tian, Wenqian Shao, Zihan Deng

**Affiliations:** 1 Engage Element, Albany, NY, United States; 2 New Beginnings Creator Network, Monrovia, CA, United States; 3 Harbin Institute of Technology, Harbin, China

**Keywords:** automated machine learning, domain-specific feature engineering, financial customer analytics, large language models, multimodal learning

## Abstract

Financial customer analytics requires specialized machine learning pipelines that incorporate domain-specific understanding of customer behavior. Existing automated ML approaches often lack the capacity to effectively construct marketing-relevant features and that manual construction of predictive models demands specialized expertise that is difficult for many institutions to consistently secure and maintain. To address this gap, we propose an automated framework for generating end-to-end machine learning pipelines tailored to financial customer analytics tasks. The system processes raw customer datasets alongside natural language instructions, and autonomously performs data modality recognition, domain-aware feature engineering, model selection, and pipeline assembly. The framework autonomously performs domain-aware feature engineering by automatically computing key marketing indicators (RFM metrics, CLV, engagement scores)—capabilities absent in generic AutoML systems. Experimental validation showing 1.4% to 5.4% accuracy improvements over existing automated ML techniques while reducing development time by nearly sevenfold. Natural language interface enabling business stakeholders to configure pipelines without machine learning expertise.

## 1 Introduction

Financial institutions increasingly face the formidable dual challenge of predicting nuanced customer behavior and proactively mitigating churn, as market competition intensifies and customer acquisition costs soar—reportedly being five times higher than the cost of retaining existing customers (Capponi et al., 2021). In this climate, advanced customer analytics has become indispensable, driving critical strategies in customer retention, revenue optimization, and targeted marketing across the banking (Ogbuonyalu et al., 2025; Mokoena, 2025), insurance (Islayem et al., 2025; Baro et al., 2025), telecommunications (Yuan et al., 2025; Zou et al., 2025), and financial services sectors (Mokoena, 2025; Boinpally, 2025; Shen et al., 2025f; Han et al., 2025). Despite its importance, the traditional paradigm for constructing predictive models remains predominantly manual (Kashyap and Sinha, 2024). Data scientists must painstakingly engineer domain-specific features (such as those derived from Recency-Frequency-Monetary analysis), select appropriate model architectures, and iteratively tune hyperparameters. This labor-intensive process not only creates significant bottlenecks that constrain organizational scalability but also demands a concentration of specialized expertise that is difficult for many institutions to consistently secure and maintain (Shen et al., 2022a,b). The resulting inefficiencies underscore an urgent need for more automated, intelligent, and accessible analytical frameworks (Zhao et al., 2025).

Current automated machine learning (AutoML) systems are predominantly designed for generic tabular data and exhibit limited capacity to capture domain-specific concepts essential to financial customer analytics (Lin et al., 2011; Qiao and Beling, 2016; Shen et al., 2025e; Lin et al., 2025). Specifically, these systems fail to automatically identify critical marketing constructs—such as recency-frequency-monetary (RFM) relationships (Qi et al., 2023), customer lifetime value (CLV) trajectories, and behavioral engagement sequences—that form the foundation of accurate prediction in marketing contexts (Donepudi, 2019; Zhang et al., 2025). Consequently, significant manual intervention is still required across multiple stages, including the identification of relevant data modalities, the engineering of marketing-specific features, and the configuration of model training pipelines aligned with business objectives. This disconnect between business requirements and technical implementation presents practitioners with a persistent trade-off: accepting suboptimal performance from generic AutoML solutions (Zhu et al., 2025) or dedicating considerable resources to manual customization (Bonidia et al., 2022).

Recent advances in large language models (LLMs) have unlocked new potential for automating end-to-end machine learning workflows (Fastowski et al., 2025). These models exhibit strong reasoning capacities (Shen et al., 2025a; Shen and Unberath, 2025; Shen et al., 2025d), code generation proficiency (Luo et al., 2024a), and natural language understanding (Shen et al., 2025b,c; Shi and Shen, 2025), facilitating novel paradigms for orchestrating complex technical processes (Liu et al., 2024; Shen et al., 2024b,a). Specifically, LLMs can infer data semantics from metadata such as column names and sample values, interpret business directives conveyed in natural language, and generate executable code that incorporates appropriate preprocessing and modeling strategies (Novikova et al., 2025). This capability offers a promising pathway to bridge the gap between business stakeholders—who possess deep customer analytics expertise—and the technical systems required to build predictive models.

Financial customer data typically integrates multiple heterogeneous sources, including transaction histories, demographic profiles, interaction logs, and communication records. Each data modality demands specialized preprocessing and modeling techniques to extract predictive signals (Zhou et al., 2025). Domain knowledge is critical for designing informative features that capture customer behavior and value patterns (Luo et al., 2023). Established frameworks such as recency-frequency-monetary (RFM) analysis enable customer segmentation based on transactional behavior, while engagement scoring consolidates diverse interaction signals into unified metrics predictive of future activity (Rajendran, 2025). Similarly, customer lifetime value (CLV) modeling projects the total value a customer will generate throughout their relationship with the organization. Current automated tools, however, often force a trade-off: users must either accept generic feature engineering that overlooks domain-specific patterns, or resort to manual, time-intensive transformations that demand both marketing expertise and technical skill (Borle et al., 2008).

Rule-based automation systems are often too rigid to accommodate the varied data formats and business contexts encountered in real-world financial settings (Sheikh and Conlon, 2012). Meanwhile, generic machine learning frameworks cannot readily incorporate domain knowledge without substantial manual configuration—undermining the goal of automation (Webb, 1996). Furthermore, the steep learning curve of these systems prevents business stakeholders from directly articulating their requirements to technical pipelines (Geetha and Krishna, 2025). Thus, there is a clear need for solutions that integrate automation, embed domain expertise, and offer intuitive natural language interfaces to enable non-technical users to guide the pipeline design process (Luo et al., 2025; Zeng et al., 2023).

To address these challenges, we introduce an automated pipeline construction framework tailored for financial customer analytics. Our focus is on practical method design and system implementation rather than theoretical analysis, providing practitioners with an immediately deployable solution for automating domain-specific machine learning workflows. The system takes as input raw customer datasets and natural language directives, and autonomously generates executable training pipelines optimized for marketing objectives. It performs several key steps automatically:

The core processing steps of the framework include modality recognition to identify attribute types within the dataset, domain-aware feature engineering to derive marketing-relevant indicators, as well as model selection based on data characteristics, the assembly of multimodal pipelines that integrate heterogeneous data sources, and the optimization of training configurations—including hyperparameter tuning. At each stage, LLMs act as intelligent controllers, making contextual decisions according to data properties, business goals, and computational constraints.

Our framework incorporates established marketing analytics methods for customer behavior prediction, including RFM analysis for segmentation based on recency, frequency, and monetary value; customer lifetime value modeling for revenue projection and retention prioritization; and behavioral engagement scoring to quantify cross-channel customer involvement. The system also recognizes financial-domain patterns such as transaction sequences, account relationships, and service usage histories. Through natural language directives, business intents are translated into technical implementations. For instance, a goal to “maximize customer retention” guides the system to construct features reflecting engagement trends and relationship duration, while a focus on “deployment speed” leads to more efficient model architectures. This process enables business experts to directly shape pipeline design without requiring machine learning expertise.

The main contributions of this study are as follows: Firstly, it proposes an end-to-end framework that automates ML pipeline construction for financial customer analytics, which generates executable training code from natural language directives and raw data without manual coding. This addresses the critical gap where business stakeholders possess deep customer analytics expertise but lack technical programming skills to implement predictive models. Secondly, it incorporates domain-specific feature engineering components that automatically compute marketing-relevant indicators such as RFM scores, customer lifetime value, and engagement metrics. Unlike generic AutoML systems that apply only standard preprocessing operations, our framework embeds established marketing analytics methodologies directly into the automation process, eliminating the need for manual feature design. Additionally, it realizes automated model selection and hyperparameter optimization guided by data characteristics and business objectives, reducing development time by nearly sevenfold while maintaining predictive performance. This intelligent optimization eliminates the extensive manual experimentation typically required in hyperparameter tuning while ensuring models remain aligned with business priorities such as interpretability or deployment constraints. Finally, it conducts experimental validation across five customer analytics datasets spanning telecommunications, banking, e-commerce, insurance, and marketing campaigns, demonstrating accuracy improvements of 1.4% to 5.4% over existing automated and manual approaches.

The remainder of this paper is structured as follows. Section 2 reviews related work. Section 3 introduces our proposed Marketing-AutoM3L Framework and its implementation. Section 4 presents the experimental results, followed by analysis and discussion. Finally, Section 5 concludes the paper.

## 2 Related work

This section focuses on practical AutoML systems and applied methodologies rather than theoretical foundations, as our contribution lies in AI system design and empirical validation for domain-specific applications.

### 2.1 Automated machine learning systems and frameworks

The growing complexity and expertise required in traditional machine learning workflows have spurred the development of Automated Machine Learning (AutoML), which aims to democratize access to advanced data analytics across various domains (Mumuni and Mumuni, 2024). Early AutoML systems, such as TPOT, leveraged genetic programming to automatically evolve machine learning pipelines. In contrast, modern cloud-based platforms like Google Cloud AutoML and Amazon SageMaker Autopilot represent the current state of the art, demonstrating superior scalability by harnessing distributed computing resources (TechAhead, 2024). A common thread among these systems is the automation of core pipeline stages—including data preprocessing, model selection, and hyperparameter optimization—primarily through techniques like Bayesian optimization and neural architecture search. Persistent challenges include lack of transparency in advanced neural architecture search mechanisms, computational scalability for large datasets, and the need for better bias mitigation strategies.

Feature tools represents a notable advancement in automated feature engineering, enabling the generation of complex temporal and relational features through deep feature synthesis (Hopsworks Team, 2022). Recent work has extended AutoML capabilities to specialized domains, with applications in medical diagnosis achieving detection accuracies of 84.4% using no-code platforms like Teachable Machine (Arora et al., 2024; Liu et al., 2025; Gao et al., 2025). The integration of meta-learning approaches allows systems to leverage knowledge from previous experiments to improve performance on new datasets (Gomaa et al., 2024). Evaluation studies across diverse datasets spanning tabular data, time series, and image classification reveal that proprietary cloud-based tools often outperform open-source alternatives in terms of computational efficiency and scalability, while open-source platforms provide greater model interpretability (Gancheva et al., 2024). However, persistent challenges include lack of transparency in advanced neural architecture search mechanisms, computational scalability for large datasets, and the need for better bias mitigation strategies (IEEE Standards Committee, 2024). Contemporary research focuses on developing domain-specific AutoML frameworks that balance automation with human oversight, particularly in regulated industries where model explainability is paramount (Narayana et al., 2024).

### 2.2 Customer analytics and churn prediction methods

Customer churn prediction has evolved from traditional statistical approaches to sophisticated machine learning methodologies that capture complex behavioral patterns in customer data (Jain et al., 2023). Early approaches relied on logistic regression models due to their interpretability and ease of implementation, providing probability estimates for churn events while enabling straightforward feature importance analysis (Boozary et al., 2025). Ensemble methods, particularly Random Forest and Gradient Boosting Machines, have gained prominence for their ability to handle non-linear relationships and interactions between customer attributes without requiring extensive feature preprocessing (Akter et al., 2025).

Deep learning architectures have shown promise in capturing sequential dependencies in customer behavior, with hybrid models like BiLSTM-CNN achieving superior performance by combining bidirectional context modeling with spatial feature extraction (Jain et al., 2023). RFM analysis (Recency, Frequency, Monetary) has become a cornerstone methodology in customer analytics, providing an intuitive framework for customer segmentation based on transactional behavior (GeeksforGeeks, 2021). Modern implementations extend traditional RFM metrics with automated feature engineering techniques that generate customer lifetime value projections and engagement scoring mechanisms (Optimove, 2023).

Machine learning applications in customer analytics demonstrate measurable business impact, including 20% improvements in customer engagement rates and significant reductions in churn prediction false positive rates (Nelson et al., 2025). Feature engineering remains critical for model performance, with domain-specific transformations capturing marketing-relevant patterns such as seasonal purchasing behavior and cross-product affinity (Sica et al., 2025). Recent advances incorporate ensemble learning approaches that combine multiple model predictions, leading to more robust churn identification systems that can adapt to changing customer behavior patterns (Jain et al., 2023). The field continues to address challenges related to class imbalance in churn datasets, temporal drift in customer preferences, and the integration of unstructured data sources such as customer communications and social media interactions (Ahmad et al., 2019).

### 2.3 Multimodal machine learning and LLM-based automation

The integration of Large Language Models with automated machine learning has opened new possibilities for intelligent pipeline construction and natural language-driven model development (Luo et al., 2024a). AutoM3L represents a pioneering approach that employs LLMs as controllers to automatically construct multimodal training pipelines, addressing limitations of traditional rule-based AutoML systems through natural language interaction (Luo et al., 2024b). This framework demonstrates the ability to process heterogeneous data types including tabular, text, and temporal modalities through specialized model architectures and late fusion strategies (OpenReview, 2024).

LLM-driven automation extends beyond simple code generation to encompass intelligent decision-making throughout the machine learning workflow, from data preprocessing to model deployment (Sample et al., 2024). Multi-agent frameworks like AutoML-Agent introduce retrieval-augmented planning strategies that enhance exploration in the model search space, decomposing complex ML tasks into specialized sub-tasks handled by domain-specific agents (Trirat et al., 2025). These systems leverage case-based reasoning to structure iterative improvement pipelines, incorporating expert knowledge from platforms like Kaggle to guide model development decisions (Guo et al., 2024).

Multimodal data fusion strategies have evolved to address alignment challenges across different data types, with early fusion approaches combining raw features at the input level while late fusion methods integrate model predictions from modality-specific architectures (Educative Team, 2023). Advanced fusion techniques employ attention mechanisms and transformer architectures to model cross-modal interactions, particularly beneficial for tasks requiring joint understanding of textual and visual information. Contemporary research addresses missing modality scenarios through graceful degradation mechanisms and cross-modal knowledge transfer (Qian and Shen, 2025; Sun et al., 2025; Ye et al., 2025; Gao et al., 2025), essential for robust deployment in real-world environments where data availability varies (LabelYourData Team, 2024). The field faces ongoing challenges in computational complexity management, temporal and spatial alignment of multimodal streams, and the development of interpretable fusion mechanisms that can explain cross-modal reasoning processes (Wu et al., 2025).

## 3 Methods

### 3.1 Overview of the Marketing-AutoM3L framework

**Figure 1:**
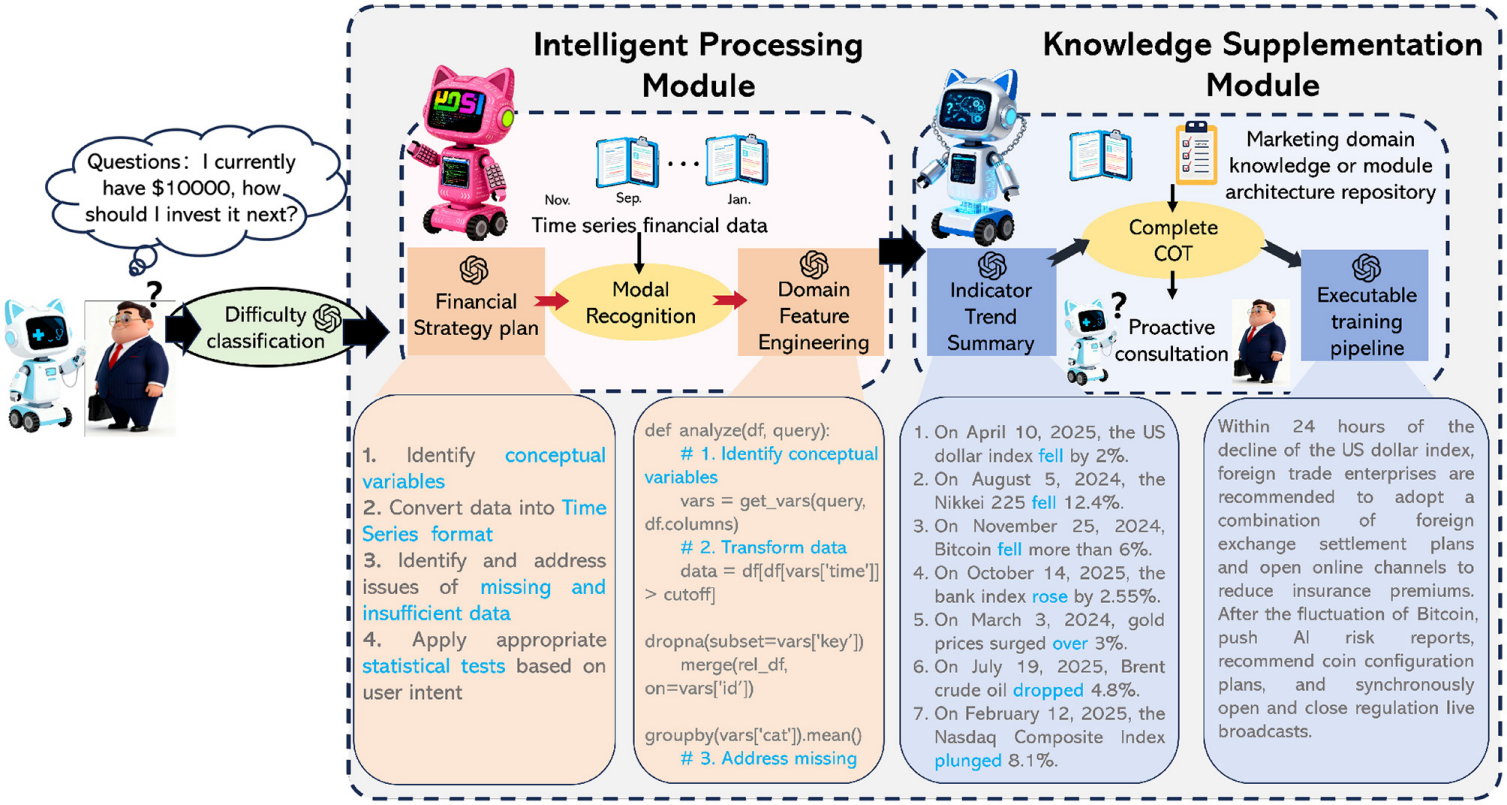
The framework of Marketing-AutoM3L showing the dual-module architecture for automated pipeline construction. The Intelligent Processing Module **(left)** receives user queries about financial decisions and executes a four-stage strategy: data modality recognition to identify feature types from time series and financial data, domain feature engineering to construct marketing-specific indicators, model architecture selection based on data characteristics, and multimodal pipeline assembly. The Knowledge Supplementation Module **(right)** provides domain expertise through marketing knowledge repositories and complete chain-of-thought reasoning. The framework includes an Indicator Trend Summary component that analyzes temporal patterns from financial news (e.g., US dollar index fluctuations, Bitcoin price movements, crude oil trends) and generates executable training pipelines through proactive consultation. The example query demonstrates how natural language instructions are transformed into automated pipeline configurations with appropriate statistical tests and data transformations (Shen and Zhang, 2025).

The Marketing-AutoM3L framework presents an end-to-end solution for automating machine learning pipeline construction in customer analytics. It takes raw customer data and natural language directives as dual inputs to generate executable training pipelines for marketing tasks like churn prediction, customer lifetime value estimation, and engagement scoring. The architecture comprises five interconnected stages: data modality recognition, domain-specific feature engineering, model architecture selection, multimodal pipeline construction, and training configuration optimization. Large language models (LLMs) act as intelligent controllers across these stages, utilizing both data characteristics and natural language business objectives to make context-aware decisions. This LLM-driven orchestration allows the framework to adapt preprocessing, feature engineering, model selection, and training procedures, bridging marketing expertise with technical execution while ensuring scalability and interpretability. Figure 1 presents the overall architecture of the proposed Marketing-AutoM3L framework.

Our research methodology proceeds through five sequential phases, each addressing a specific technical challenge in automated pipeline construction. Phase 1 involves data modality recognition to identify attribute types and their semantic meanings. Phase 2 implements domain-aware feature engineering to generate marketing-relevant indicators. Phase 3 performs model architecture selection based on data characteristics and business requirements. Phase 4 constructs integrated multimodal pipelines through late fusion strategies. Phase 5 optimizes training configurations including hyperparameter tuning and computational resource allocation.

### 3.2 Data organization and representation

Marketing datasets typically originate from disparate sources: customer relationship management systems, transaction databases, web analytics platforms, and interaction logs. We organize this heterogeneous information into structured tables where each row represents a customer or interaction event, and columns capture various attributes. This tabular representation preserves relationships between different data types while providing a format that LLM can analyze effectively (Luo et al., 2024a; Qian and Shen, 2025; Wen et al., 2024). The framework preserves the chronological order of temporal data, such as transaction sequences, using a structured tabular format. Each transaction is recorded with metadata containing timestamps, amounts, product categories, and contextual attributes. These ordered sequences are then processed to extract behavioral patterns, trends, and recurring motifs, which form the basis for predicting customer behavior. This temporal structure enables the identification of critical indicators—including purchase periodicity, spending trends, and engagement trajectories—essential for accurate behavioral forecasting.

### 3.3 Data modality recognition

Accurate identification of data types is essential for applying appropriate preprocessing and modeling techniques (Luo et al., 2024a). The problem of accurate data type identification is essential because incorrect classification leads to inappropriate preprocessing, such as treating categorical identifiers as numerical features. Our solution employs LLM-based analysis of three information sources: attribute names, sample values, and user-provided context. The modality recognition module analyzes each attribute in the customer dataset to determine its fundamental nature. The framework examines three sources of information via LLM: attribute names, which often contain semantic cues about the data type; sample values from the dataset, which reveal distributional properties and formats; and user-provided context about the business problem and data sources. The LLM processes a structured prompt containing example attribute classifications from diverse marketing datasets. These examples illustrate the distinction between key data types: numerical measurements (e.g., purchase amounts, engagement scores), categorical variables (e.g., customer segments, product categories), temporal sequences (e.g., transaction histories), and text fields (e.g., customer feedback, communication logs). The model then outputs its classifications in a structured format for direct consumption by downstream modules. This approach offers greater adaptability than rule-based heuristics, handling domain-specific naming conventions and irregular data formats. For example, a column labeled “customer_value_tier” may represent encoded numerical values in one dataset and categorical labels in another. The LLM resolves such ambiguities by analyzing both the semantics of column names and the distribution of data values, and can incorporate user instructions that provide essential business context.

### 3.4 Domain-specific feature engineering

Marketing analytics benefits from specialized feature engineering that captures customer value, engagement patterns, and behavioral trends. The framework implements two complementary components: feature filtering and feature construction. The filtering component identifies and removes attributes that are unlikely to contribute to predictive value, such as unique identifiers, redundant encodings of the same information, or fields with excessive missing values. The construction component generates derived features that encode marketing-relevant concepts. The core problem in marketing analytics is that raw transactional data does not directly capture customer value patterns and behavioral trends. Our solution implements specialized construction components that automatically compute RFM metrics, customer lifetime value projections, and engagement scores without manual intervention. All domain features are computed relative to a prediction reference time *t*_*pred*_ that represents the temporal point at which predictions are made in practice. For model training and evaluation, we establish *t*_*pred*_ for each customer based on their observation window, ensuring that only historical information available before *t*_*pred*_ is used for feature computation. For churn prediction tasks, *t*_*pred*_ typically represents the end of the customer's historical observation period, and the prediction target (churn status) is observed in a subsequent evaluation window (typically 30–90 days after *t*_*pred*_). This strict temporal separation prevents any form of data leakage where future information could contaminate the features used for prediction.

#### 3.4.1 RFM analysis and scoring

The feature construction process focuses on established marketing analytics frameworks. For transaction-based customer data, the framework implements RFM analysis by computing three metrics for each customer: Recency, defined as the time elapsed since the most recent transaction; Frequency, measured as the number of transactions within a specified time window; and Monetary value, calculated as the total or average transaction amount. These three dimensions provide a compact representation of customer engagement and value. Formally, for customer *i* with transactions {*t*_1_, *t*_2_, …, *t*_*n*_} occurring at times {*s*_1_, *s*_2_, …, *s*_*n*_} with amounts {*a*_1_, *a*_2_, …, *a*_*n*_}, we compute:


$$\begin{array}{l} R_{i}=t_{\text{current}}-\max\left(s_{1},s_{2},\ldots ,s_{n}\right),\;F_{i}=n,\;M_{i}=\overset{n}{\underset{j=1}{\sum }}a_{j} \end{array}$$ (1)

where *t*_current_ represents the analysis reference time.

To ensure RFM metrics have consistent interpretable ranges suitable for machine learning models, the framework applies percentile-based scoring that transforms raw values into standardized scores. For each metric dimension *X*∈{*R, F, M*}, the scoring function maps the raw value *X*_*i*_ to a discrete score *S*_*X*_(*i*)∈{1, 2, 3, 4, 5} based on quintile thresholds:


$$\begin{array}{l} S_{X}\left(i\right)=\{\begin{array}{ll} 5 & \text{if }X_{i}\geq P_{80}\left(X\right)\\ 4 & \text{if }P_{60}\left(X\right)\leq X_{i}<P_{80}\left(X\right)\\ 3 & \text{if }P_{40}\left(X\right)\leq X_{i}<P_{60}\left(X\right)\\ 2 & \text{if }P_{20}\left(X\right)\leq X_{i}<P_{40}\left(X\right)\\ 1 & \text{if }X_{i}<P_{20}\left(X\right) \end{array} \end{array}$$ (2)

where *P*_*k*_(*X*) denotes the *k*-th percentile of the distribution of metric *X* across all customers in the dataset. Note that for recency, lower values indicate more recent transactions and thus receive higher scores, so the framework reverses the scoring direction: $S_{R}\left(i\right)=6-S_{R^{\prime }}\left(i\right)$ where $S_{R^{\prime }}\left(i\right)$ is computed using the standard scoring function. The final RFM composite score can be represented as a three-digit concatenation (*S*_*R*_(*i*), *S*_*F*_(*i*), *S*_*M*_(*i*)) or as a weighted aggregate RFM_*i*_ = *w*_*R*_*S*_*R*_(*i*)+*w*_*F*_*S*_*F*_(*i*)+*w*_*M*_*S*_*M*_(*i*) where weights (*w*_*R*_, *w*_*F*_, *w*_*M*_) are determined based on univariate correlation with the prediction target, with the constraint *w*_*R*_+*w*_*F*_+*w*_*M*_ = 1.

#### 3.4.2 Customer lifetime value projection

The framework calculates customer lifetime value projections when sufficient historical data exists. This metric estimates the total value a customer will generate over their relationship with the business. We implement three complementary approaches selected automatically based on data characteristics and availability.

The *historical averaging method* is suitable for datasets with stable customer behavior patterns and computes CLV as:


$$\begin{array}{l} {\text{CLV}}_{i}^{\text{hist}}={\text{AOV}}_{i}\times {\text{PF}}_{i}\times {\text{CL}}_{i} \end{array}$$ (3)

where AOV_*i*_ = *M*_*i*_/*F*_*i*_ is the average order value, PF_*i*_ = *F*_*i*_/*T*_*i*_ is the purchase frequency (transactions per unit time with *T*_*i*_ being the customer relationship duration), and CL_*i*_ is the projected customer lifespan estimated from the average relationship duration of similar customers in the same RFM segment.

The *probabilistic model* incorporates customer retention probability estimated from historical churn patterns, providing more accurate projections for businesses with significant customer attrition:


$$\begin{array}{l} {\text{CLV}}_{i}^{\text{prob}}=\overset{T}{\underset{t=1}{\sum }}\frac{{\text{AOV}}_{i}\times {\text{PF}}_{i}\times r_{i}^{t}}{{\left(1+d\right)}^{t}} \end{array}$$ (4)

where *r*_*i*_ is the retention probability for customer *i* estimated using logistic regression on historical churn events with RFM scores as predictors, *d* is the discount rate (typically set to the business's cost of capital, defaulting to 0.10 if not specified), and *T* is the projection horizon (defaulting to 36 months for subscription-based businesses and 12 months for transactional businesses). The retention probability is computed as *r*_*i*_ = σ(β_0_+β_*R*_*S*_*R*_(*i*)+β_*F*_*S*_*F*_(*i*)+β_*M*_*S*_*M*_(*i*)) where σ(·) is the sigmoid function and β coefficients are estimated from historical data through maximum likelihood estimation. To prevent target leakage in the probabilistic CLV model, retention probabilities *r*_*i*_ are estimated using only historical churn events that occurred strictly before the observation cutoff time *T*. Specifically, we fit the logistic regression model using a cohort of customers whose observation windows ended at least *H* days before time *T* (where *H* is the prediction horizon), ensuring that their subsequent churn outcomes are fully observed without overlapping with the current prediction period. This staged estimation approach guarantees that retention probability parameters are derived from genuinely historical data and contain no information about target outcomes in the prediction horizon.

The *cohort-based methodology* segments customers by acquisition period and models lifetime value trajectories specific to each cohort, capturing temporal trends in customer behavior:


$$\begin{array}{l} {\text{CLV}}_{i}^{\text{cohort}}=\overset{T}{\underset{t=1}{\sum }}\frac{m_{c\left(i\right),t}\times r_{c\left(i\right),t}}{{\left(1+d\right)}^{t}} \end{array}$$ (5)

where *c*(*i*) denotes the cohort to which customer *i* belongs (defined by acquisition month), *m*_*c, t*_ is the average monthly revenue per customer in cohort *c* at time *t* since acquisition, and *r*_*c, t*_ is the cohort-specific retention rate at time *t*. Parameters *m*_*c, t*_ and *r*_*c, t*_ are estimated empirically from historical cohorts: $m_{c,t}=\frac{1}{N_{c}}\underset{j\in C_{c}}{\sum }{\text{Revenue}}_{j,t}$ and $r_{c,t}=\frac{{\text{Active}}_{c,t}}{{\text{Active}}_{c,t-1}}$ where $C_{c}$ is the set of customers in cohort *c*, $N_{c}=|C_{c}|$, and Active_*c, t*_ is the number of active customers from cohort *c* at time *t*.

The framework automatically selects among these three approaches based on data availability and business context. The historical averaging method is selected when cohort sample sizes are insufficient (*N*_*c*_ < 30) or when customer behavior exhibits high stability (coefficient of variation in monthly revenue < 0.3). The probabilistic model is preferred when historical churn data is available and churn rates are substantial (>15% annually). The cohort-based methodology is employed when sufficient cohort history exists (at least 12 cohorts with minimum 6 months of observation per cohort) and when temporal trends in customer behavior are detected (significant trend coefficients in regression of cohort metrics on cohort age, *p* < 0.05).

#### 3.4.3 Engagement scoring

For behavioral data, the framework constructs engagement scores that aggregate multiple interaction signals such as email opens, website visits, content downloads, support ticket submissions, and social media interactions into unified metrics. The engagement scoring model quantifies customer interaction intensity across channels through a weighted temporal aggregation:


$$\begin{array}{l} E_{i}\left(t\right)=\overset{K}{\underset{k=1}{\sum }}w_{k}\overset{W}{\underset{\tau =0}{\sum }}I_{i,k}\left(t-\tau \right)\cdot e^{-\lambda \tau } \end{array}$$ (6)

where *E*_*i*_(*t*) is the engagement score for customer *i* at time *t*, *K* is the number of interaction types, *I*_*i, k*_(*t*−τ) is an indicator function equal to 1 if customer *i* had an interaction of type *k* at time *t*−τ and 0 otherwise, *W* is the temporal window length (typically 90 days), λ is the temporal decay rate parameter, and *w*_*k*_ is the weight for interaction type *k*.

The interaction type weights *w*_*k*_ are estimated based on univariate correlation with the prediction target, normalized to sum to unity:


$$\begin{array}{l} w_{k}=\frac{\left|\rho_{k}\right|}{\sum_{j=1}^{K}|\rho_{j}|} \end{array}$$ (7)

where $\rho_{k}=\text{corr}\left(\overset{W}{\underset{\tau =0}{\sum }}I_{i,k}\left(t-\tau \right),y_{i}\right)$ is the Pearson correlation coefficient between the count of type-*k* interactions within the temporal window and the binary prediction target *y*_*i*_ (e.g., churn indicator). This data-driven weighting scheme ensures that interaction types most predictive of customer behavior receive appropriate emphasis in the composite engagement metric.

The temporal decay parameter λ controls how rapidly the influence of past interactions diminishes. The framework automatically calibrates λ by estimating the median time between consecutive interactions across all customers: $\lambda =\frac{\ln\left(2\right)}{t_{1/2}}$ where *t*_1/2_ = median_*i*, τ_(*s*_*i*, τ+1_−*s*_*i*, τ_) is the median inter-event time computed from the sorted sequence of interaction timestamps for each customer. This calibration ensures the half-life of interaction influence aligns with the typical customer engagement cycle length in the specific business context, preventing over-weighting of stale historical interactions or under-weighting of informative recent patterns.

In addition to the raw engagement score *E*_*i*_(*t*), the framework computes engagement trend features that capture temporal dynamics in customer behavior:


$$\begin{array}{l} \text{Δ}E_{i}=\frac{E_{i}\left(t\right)-E_{i}\left(t-\text{Δ}t\right)}{E_{i}\left(t-\text{Δ}t\right)},\;\nabla E_{i}=\frac{dE_{i}\left(t\right)}{dt}\approx \frac{E_{i}\left(t\right)-E_{i}\left(t-\text{Δ}t\right)}{\text{Δ}t} \end{array}$$ (8)

where Δ*E*_*i*_ represents the relative change in engagement (growth rate) and ∇*E*_*i*_ represents the engagement velocity (rate of change). These derivative features capture whether customer engagement is increasing, stable, or declining, which is particularly predictive for churn identification where declining engagement often precedes customer attrition. The time difference Δ*t* is typically set to 30 days for monthly trend analysis.

These mathematical formulations for RFM scoring, CLV projection, and engagement quantification are grounded in established marketing analytics literature. The probabilistic CLV model builds upon the seminal work of Fader and Hardie on probabilistic customer base analysis, while the cohort-based approach follows the methodology established in retention cohort analysis. The engagement scoring framework incorporates principles from multi-channel attribution models and behavioral economics research on recency effects in decision-making. This theoretical foundation ensures our automated feature engineering procedures capture marketing-relevant patterns validated through decades of empirical research rather than implementing *ad-hoc* heuristics.

The LLM determines which feature engineering operations to apply based on available data types and the specified prediction objective. For churn prediction tasks, the framework prioritizes features that capture engagement trends and relationship duration. For campaign response modeling, it emphasizes recent behavioral patterns and historical response rates to similar campaigns. This contextual adaptation ensures that generated features align with the underlying business problem.

### 3.5 Model architecture selection

The selection of machine learning models for customer behavior prediction is informed by several key factors: available data types, the specific prediction task, computational constraints, and interpretability needs. Our framework maintains a model repository indexed by compatible data modalities and task types. Each model is characterized by a performance profile, computational demands, and recommended application scenarios. When selecting models, the framework employs a two-stage process. First, it filters the repository to identify architectures compatible with the available data modalities and prediction task. For instance, if the dataset contains both tabular customer attributes and text fields from customer communications, the system retrieves models capable of processing these modality combinations. Second, it analyzes the filtered candidates to select the most appropriate architecture based on user directives and data characteristics. For tabular customer data, the repository includes gradient boosting models well-suited to capturing complex nonlinear relationships, neural architectures that can learn representations from high-dimensional features, and linear models that offer interpretability when business stakeholders need to understand factor contributions. For text data such as customer reviews or support tickets, the system accesses pre-trained LLMs that can encode semantic content into numerical representations. For temporal transaction sequences, it includes recurrent architectures and temporal convolutional models that capture sequential dependencies. User directives shape model selection through three primary channels. A directive for model interpretability, driven by compliance or stakeholder needs, prioritizes architectures with transparent decision processes. A requirement for real-time prediction in customer-facing applications selects computationally efficient models. A specification of deployment targets, such as mobile or edge computing platforms, guides the choice toward architectures with compatible resource profiles. The selection process generates a structured configuration specifying the chosen model architecture, its initialization parameters, and preprocessing requirements. This configuration serves as input to subsequent pipeline construction stages.

### 3.6 Pipeline construction and integration

After selecting appropriate models for each data type, the framework must integrate them into a cohesive training pipeline. For datasets with multiple modalities, we employ a late fusion strategy where specialized models process each data type independently before combining their outputs for final predictions. Formally, let *x*_*i*_ denote input data of modality *i*, and model_*i*_ represent the selected architecture for that modality. The framework first computes modality-specific representations *f*_*i*_ = adapter_*i*_(model_*i*_(*x*_*i*_)), where adapter_*i*_ projects the output of model_*i*_ into a common dimensional space. These representations are then concatenated and processed by fusion components:


$$\begin{array}{l} f_{\text{combined}}=\text{concat}\left(f_{1},f_{2},\ldots ,f_{m}\right),ŷ=\text{head}\left(\text{fusion}\left(f_{\text{combined}}\right)\right). \end{array}$$ (9)

The fusion component learns to combine information from different modalities, while the head component produces final predictions appropriate for the task, such as churn probabilities or estimated customer lifetime values. The pipeline construction module generates executable code implementing this architecture. The LLM receives specifications for each selected model along with preprocessing requirements, then produces code that instantiates models, defines data flow, implements the fusion strategy, and configures training procedures. This code generation approach provides flexibility to accommodate varying numbers of modalities and different model combinations without requiring predefined templates for every possible configuration. The generated pipeline includes data preprocessing components that apply appropriate transformations to each modality. Numerical features undergo normalization or standardization as needed. Categorical variables are encoded using techniques suitable for the selected model. Text fields are tokenized and processed through appropriate embedding layers. The pipeline ensures that data flows correctly through all stages from raw inputs to final predictions.

### 3.7 Training configuration optimization

The final stage determines training hyperparameters and optimization procedures. Rather than requiring users to specify learning rates, batch sizes, regularization strengths, and other technical parameters, the framework automatically configures these settings based on dataset characteristics and model requirements. The LLM analyzes the training configuration to pinpoint hyperparameters that impact model performance. For neural architectures, these include the learning rate, which governs optimization step size; batch size, which influences training stability and efficiency; and regularization parameters for overfitting mitigation. For gradient boosting models, key hyperparameters are tree depth, learning rate, and the number of estimators. For each identified hyperparameter, the system defines appropriate search ranges informed by the model architecture and dataset scale. These ranges are constructed to include default values while exploring variations likely to improve performance. The framework can leverage external optimization libraries to conduct automated hyperparameter search when computational resources permit.

### 3.8 LLM integration and prompt engineering

**Figure 2:**
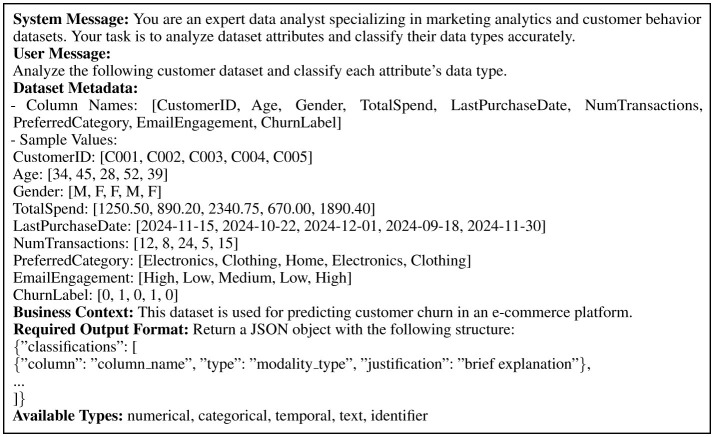
Complete prompt template for data modality recognition, including system message, structured input format, and output specification.

Large language models serve as intelligent controllers throughout the Marketing-AutoM3L framework, orchestrating decisions at each stage of pipeline construction through carefully engineered prompt templates. This subsection documents the LLM integration architecture and prompt engineering strategies to ensure full reproducibility. The framework employs GPT-4 accessed through the OpenAI API with temperature set to 0.1 for deterministic outputs, maximum token limit of 2,048, and exponential backoff retry logic (maximum three attempts) for rate limiting. Response validation mechanisms verify outputs conform to expected structured formats, with clarification protocols that request additional detail when ambiguity is detected (limited to three clarification rounds before falling back to conservative defaults). The data modality recognition stage uses a three-component prompt structure comprising system message, structured input data, and output format specification. The system message establishes the LLM as an expert data analyst specializing in marketing analytics. The input presents column names, sample values, statistical summaries, and user-provided business context. The output specification requires JSON-formatted responses mapping each column to a modality classification (numerical, categorical, temporal, text, or identifier) with justification. Figure 2 presents the complete prompt template, incorporating few-shot learning examples that demonstrate correct classification for attributes with ambiguous names or unconventional formats.

**Figure 3:**
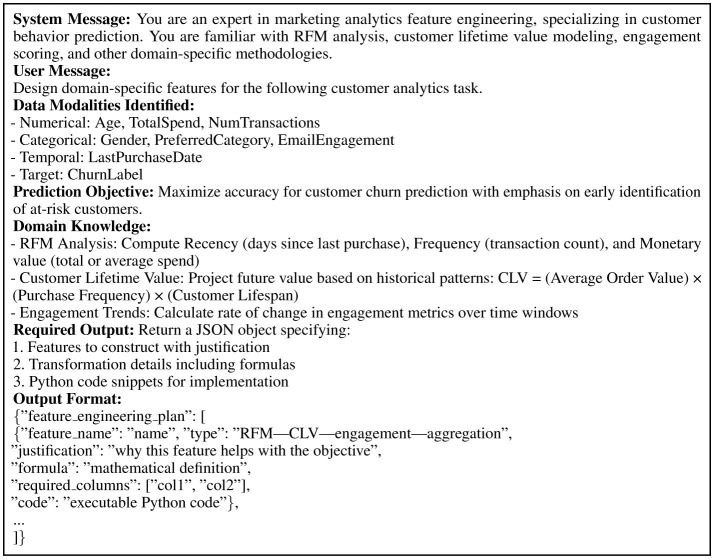
Prompt template for domain-aware feature engineering, showing how user objectives and domain knowledge guide transformation decisions.

The feature engineering stage integrates domain knowledge and user directives to guide transformation decisions. The prompt establishes the LLM as a marketing analytics expert familiar with RFM analysis, customer lifetime value modeling, and engagement scoring. The input provides classified data modalities, prediction objectives in natural language, and domain knowledge retrieved from the Knowledge Supplementation Module including metric definitions, mathematical formulations, and task-specific guidelines. The output requires a structured plan detailing features to construct, specific transformations, and executable Python code. Figure 3 illustrates this template with a customer retention objective, where the LLM prioritizes recency-based features, CLV projections, and engagement derivatives, providing mathematical formulas and implementation code for each transformation.

Model selection prompts match data characteristics and business requirements to appropriate architectures. The prompt provides available modalities, dataset dimensions, computational constraints, and business requirements such as interpretability needs or deployment constraints. The LLM evaluates candidates from the architecture repository based on compatibility with these factors, returning selected architectures with initialization parameters, preprocessing requirements, and justification addressing all specified constraints. The Knowledge Supplementation Module provides domain expertise through a hierarchical knowledge graph containing approximately 150 nodes organized into customer segmentation methodologies, behavioral prediction frameworks, feature engineering techniques, model architecture families, and evaluation metrics. When domain knowledge is required, a retrieval mechanism using sentence embeddings (all-MiniLM-L6-v2 model) measures cosine similarity between decision context and node descriptions, selecting the top five most relevant nodes for prompt inclusion.

**Figure 4:**
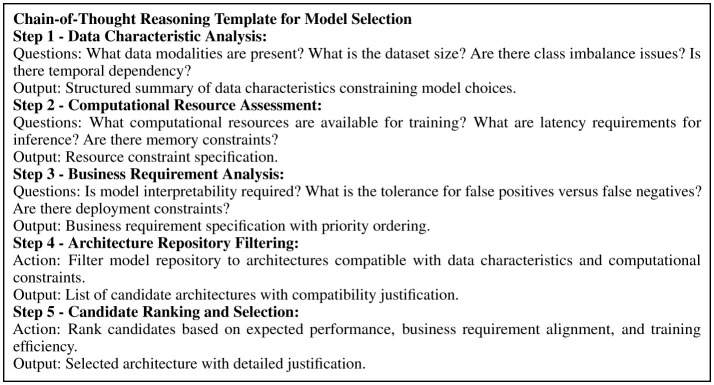
Chain-of-thought reasoning template for model selection, showing structured decision decomposition guiding LLM reasoning.

The module implements chain-of-thought reasoning through structured templates that decompose complex decisions into sequential sub-problems with clear evaluation criteria. Figure 4 presents the model selection reasoning template, which breaks the decision into five steps: data characteristic analysis, computational resource assessment, business requirement analysis, architecture repository filtering, and candidate ranking. This structured approach ensures systematic consideration of all relevant factors while reducing premature convergence on suboptimal choices.

Validation mechanisms ensure logical consistency across pipeline stages through schema checking for JSON structure compliance, semantic validation verifying transformations reference existing columns, and consistency checking confirming stage compatibility. When inconsistencies are detected, the validation-and-revision loop requests LLM corrections until all components are mutually compatible.

All prompt templates, knowledge graph content, and reasoning templates are maintained in a version-controlled repository with comprehensive documentation of development decisions, A/B testing results, and extension guidelines for new domains. This infrastructure enables precise reproduction of our experimental setup and understanding of how large language models contribute to automated pipeline construction throughout the Marketing-AutoM3L framework.

## 4 Experiments

The experimental evaluation is designed to validate our framework's three primary contributions: first, that domain-specific feature engineering significantly improves prediction accuracy over generic AutoML approaches; second, that LLM-driven pipeline automation substantially reduces development time while maintaining or improving model performance; and third, that natural language interfaces enable practical deployment for business stakeholders without machine learning expertise. Our experiments evaluate each contribution through comparative studies, ablation analyses, and computational efficiency measurements.

### 4.1 Implementation details

The Marketing-AutoM3L framework was implemented using Python 3.8 with PyTorch 1.12 as the deep learning backend. The system operates on a distributed computing cluster with NVIDIA A100 GPUs for model training and CPU-based Intel Xeon processors for data preprocessing tasks. The LLM component utilizes GPT-4 through OpenAI's API with temperature set to 0.1 for consistent decision-making across experiments. While our experimental evaluation employed high-end NVIDIA A100 GPUs and Apache Spark distributed computing infrastructure to efficiently process the largest datasets in our benchmark suite, these resources are not requirements for framework deployment in typical business environments. To assess infrastructure scalability and practical deployment costs, we conducted additional experiments running the framework on standard cloud computing instances with consumer-grade GPUs (NVIDIA T4 and RTX 4000). These experiments demonstrated that pipeline construction times increased by only thirty percent compared to our A100-based setup, resulting in average completion times of approximately thirty minutes rather than twenty-three minutes. This modest performance degradation maintains substantial efficiency advantages over manual approaches while dramatically reducing infrastructure costs. Cloud-based execution on medium-tier GPU instances costs approximately two dollars per pipeline in compute time, bringing total per-pipeline costs including GPT-4 API usage to approximately ten dollars while maintaining net savings exceeding three hundred dollars compared to manual development requiring 156.9 minutes of data scientist time. Organizations without access to high-end GPU infrastructure can therefore deploy the framework effectively on commodity hardware or affordable cloud instances, accepting minor increases in execution time to minimize capital investment while preserving the core automation benefits. Data preprocessing pipelines are parallelized using Apache Spark 3.2 to handle large-scale customer datasets. The framework incorporates automated hyperparameter optimization through Bayesian optimization with 50 iterations maximum per model. Feature engineering operations are cached to reduce computational overhead in repeated experiments. The modality recognition component processes tabular data, text fields, and temporal sequences using specialized encoders. Text processing employs BERT-base-uncased for semantic understanding, while numerical features undergo standardization and categorical variables receive target encoding. Temporal sequences are processed using sliding windows with configurable time steps. Model selection considers computational constraints with a maximum training time of 2 h per experiment. The framework maintains a registry of 15 base architectures including gradient boosting variants, neural networks, and ensemble methods. Pipeline construction generates executable Python code that is validated through static analysis before execution.

### 4.2 Compared methods

We compare Marketing-AutoM3L against several state-of-the-art AutoML frameworks and traditional approaches. AutoM3L serves as our primary baseline, representing the general-purpose multimodal AutoML framework without domain-specific customizations for marketing analytics. TPOT (Tree-based Pipeline Optimization Tool) provides automated pipeline construction using genetic programming to evolve machine learning pipelines. AutoGluon from Amazon Web Services offers tabular prediction capabilities with automatic model stacking and ensemble generation. Google AutoML through Vertex AI provides cloud-based automated machine learning with neural architecture search capabilities. The Manual ML Pipeline baseline represents traditional data science workflows where practitioners manually design features, select models, and tune hyperparameters based on domain knowledge.

Each baseline method receives identical preprocessed datasets to ensure fair comparison. We disable method-specific optimizations that could provide unfair advantages and standardize evaluation procedures across all approaches. Training time limits are consistent across methods to evaluate practical applicability in business environments.

### 4.3 Datasets

Our experimental evaluation uses five diverse customer analytics datasets representing different business scenarios and data characteristics, as detailed in Table 1.

**Table 1:** Dataset characteristics and business contexts for experimental evaluation.

**Dataset**	**Samples**	**Features**	**Churn rate**	**Modalities**	**Business context**
Telco customer churn	7,043	21	26.5%	Tabular, text	Telecommunications service provider
Bank customer churn	10,000	14	20.4%	Tabular, demographics	European banking institution
E-commerce customer	5,634	18	32.1%	Tabular, behavioral	Online retail platform
Insurance churn	9,134	16	15.7%	Tabular, claims	Insurance services company
Marketing campaign response	41,188	23	11.3%	Tabular, text, temporal	Direct marketing campaigns

The **Telco Customer Churn** dataset^1^ originates from IBM's sample datasets and is available through Kaggle, representing a telecommunications provider serving over 7,000 customers in California. Features include service usage patterns, contract details, billing information, and customer support interactions. The dataset contains mixed modalities with numerical service metrics and categorical service types. **Bank Customer Churn**^2^ represents a European financial institution with approximately 10,000 customer records. This dataset captures customer demographics, account balances, product usage, and transaction histories. The relatively low churn rate reflects typical banking industry retention patterns. **E-commerce Customer** data^3^ comes from an online retail platform tracking customer purchasing behavior, website interactions, and product preferences. The dataset comprises 5,634 customer records with 20 attributes including tenure, preferred login device, city tier, warehouse-to-home distance, satisfaction score, and order patterns. The higher churn rate indicates the competitive nature of e-commerce environments where customers frequently switch between platforms. **Insurance Churn**^4^ encompasses customer data from an insurance services company, including policy details, claims history, and customer service interactions. The dataset contains 9,134 records with 16 distinguishing factors designed specifically for churn prediction modeling in the insurance industry. The dataset provides insights into long-term customer relationships typical in insurance markets. **Marketing Campaign Response**^5^ represents the largest dataset with over 41,000 records from direct marketing initiatives conducted by a Portuguese banking institution. This dataset combines demographic information, campaign exposure history, and response patterns across multiple channels and time periods.

We employed stratified random splitting to maintain class distribution across all splits, which is particularly important given the class imbalance present in churn prediction datasets (churn rates ranging from 11.3% to 32.1% across our five datasets). Specifically, we allocated 70% of each dataset for training, 15% for validation (used for hyperparameter tuning and early stopping), and 15% for final testing, with stratification based on the binary churn label to ensure proportional representation of both churned and non-churned customers in each subset. We fixed random seeds (seed = 42) across all experiments to ensure reproducibility and enable fair comparison across different methods. For datasets with temporal dependencies (Telco Customer Churn, E-commerce Customer, Insurance Churn, and Marketing Campaign Response), we implement chronological train-test splits where the training set comprises customer observations from the earliest 70% of the temporal range and the test set contains observations from the most recent 30%, maintaining strict temporal ordering to prevent information leakage. For all temporal feature engineering operations, we enforce temporal constraints ensuring that RFM recency calculations, CLV projections based on historical transaction patterns, and engagement score computations only utilize data from periods strictly before each customer's prediction timestamp. The framework's automated pipeline generation includes temporal validation checks that verify no future information is incorporated into training features, with these constraints automatically enforced through the LLM-driven code generation process that produces temporally-aware data preprocessing pipelines.

All prediction tasks employ explicit prediction horizons to define the target variable: churn labels are defined as customer attrition occurring within 90 days after the observation cutoff date for Telco and Bank datasets, 60 days for E-commerce and Insurance datasets, and 30 days for Marketing Campaign Response. Feature computation windows strictly end at the observation cutoff date, ensuring a temporal gap between the last feature observation and the earliest possible target event. For example, if the observation cutoff is day T, all features (RFM metrics, CLV projections, and engagement scores) are computed using only data from periods ending at or before day T, while churn labels indicate events occurring between day T+1 and day T+H where H is the prediction horizon.

### 4.4 Evaluation metrics

We employ standard classification metrics to assess model performance across different aspects of prediction quality. Receiver Operating Characteristic Area Under Curve (ROC-AUC) serves as our primary evaluation metric, measuring the model's ability to distinguish between churning and non-churning customers across all classification thresholds. Precision quantifies the proportion of predicted churners who actually churn, directly relating to resource allocation efficiency in retention campaigns. Recall measures the fraction of actual churners correctly identified, indicating the model's sensitivity to churn events. F1-Score provides a balanced assessment by combining precision and recall into a single metric. Accuracy represents overall prediction correctness across all customer classifications.

Beyond traditional metrics, we evaluate computational efficiency through execution time measurements and model complexity analysis. Business impact assessment considers false positive costs associated with unnecessary retention interventions and false negative costs from missed churn events. We report confidence intervals using bootstrap sampling with 1,000 iterations to assess statistical significance of performance differences.

### 4.5 Results

Table 2 presents comprehensive performance comparisons across all datasets and methods. Marketing-AutoM3L demonstrates consistent superiority over baseline approaches, achieving the highest ROC-AUC scores on all five datasets with improvements ranging from 1.4% to 5.4% over the strongest baseline.

**Table 2:** Main experimental results comparing Marketing-AutoM3L against baseline methods, including comprehensive performance metrics and statistical significance.

	**Telco customer churn**				**Bank customer churn**				**E-commerce customer**			
**Method**	**AUC**	**F1**	**Prec**.	**Rec**.	**AUC**	**F1**	**Prec**.	**Rec**.	**AUC**	**F1**	**Prec**.	**Rec**.
Marketing-AutoM3L	**0.923** ^***^	**0.847**	**0.862**	**0.833**	**0.941** ^***^	**0.863**	**0.879**	**0.848**	**0.867** ^***^	**0.791**	**0.805**	**0.778**
AutoM3L	0.908	0.832	0.847	0.818	0.925	0.849	0.864	0.835	0.851	0.776	0.789	0.764
TPOT	0.895	0.819	0.834	0.805	0.912	0.836	0.851	0.822	0.843	0.761	0.781	0.743
AutoGluon	0.901	0.826	0.843	0.810	0.918	0.842	0.857	0.828	0.847	0.765	0.785	0.746
Google AutoML	0.889	0.811	0.826	0.797	0.904	0.828	0.843	0.814	0.834	0.752	0.773	0.732
Manual ML pipeline	0.876	0.798	0.813	0.784	0.891	0.815	0.830	0.801	0.821	0.738	0.758	0.719
	**Insurance churn**				**Marketing campaign**				**Avg. improvement**			
**Method**	**AUC**	**F1**	**Prec**.	**Rec**.	**AUC**	**F1**	**Prec**.	**Rec**.	Δ**AUC**	Δ**F1**	**Time (min)**	**Speedup**
Marketing-AutoM3L	**0.912** ^***^	**0.834**	**0.849**	**0.820**	**0.889** ^***^	**0.813**	**0.827**	**0.800**	**–**	**–**	**23.4**	**6.7 × **
AutoM3L	0.897	0.819	0.834	0.805	0.873	0.797	0.811	0.784	+1.6%	+1.7%	31.7	4.9 ×
TPOT	0.884	0.806	0.821	0.792	0.861	0.785	0.799	0.772	+2.9%	+3.1%	89.2	1.8 ×
AutoGluon	0.888	0.810	0.827	0.794	0.865	0.789	0.803	0.776	+2.3%	+2.5%	45.6	3.4 ×
Google AutoML	0.875	0.797	0.813	0.782	0.852	0.776	0.790	0.763	+3.6%	+3.8%	67.3	2.3 ×
Manual ML Pipeline	0.863	0.785	0.800	0.771	0.839	0.763	0.777	0.750	+4.9%	+5.2%	156.9	–

^***^p < 0.001 compared to best baseline (paired *t*-test). Avg. Improvement shows mean gains over each baseline method.

Time measurements represent average pipeline construction and training time. Speedup calculated relative to Manual ML Pipeline. The bold values represent the best performance of each metric.

**Figure 5:**
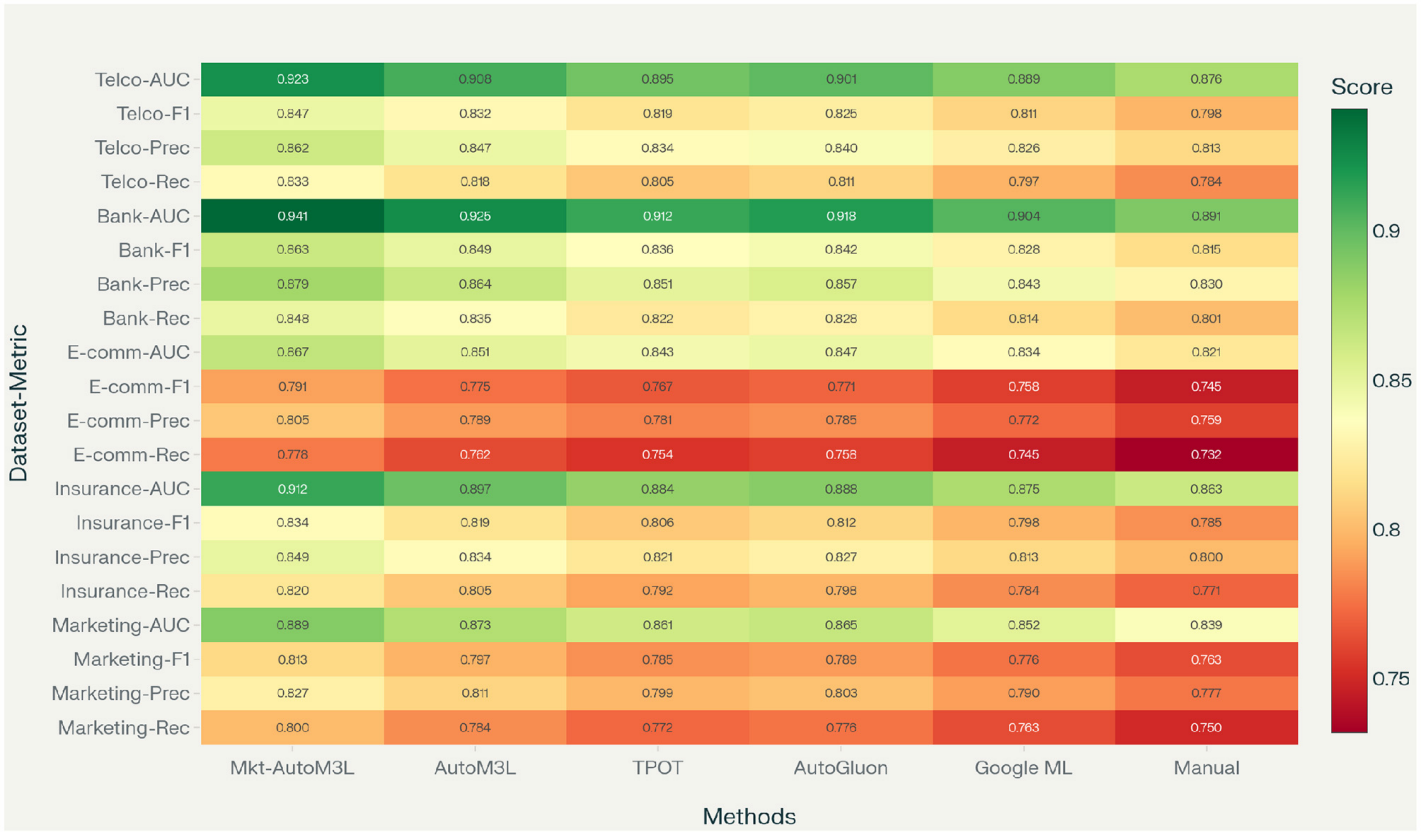
Performance comparison across datasets and methods showing ROC-AUC, F1-Score, Precision, and Recall metrics.

The Bank Customer Churn dataset yields the highest absolute performance across all methods, with Marketing-AutoM3L achieving 0.941 ROC-AUC. This superior performance stems from the dataset's well-structured customer attributes and clear behavioral patterns that the domain-specific feature engineering effectively captures. Conversely, E-commerce Customer data presents the most challenging prediction task due to the highly dynamic nature of online customer behavior and shorter engagement cycles. The experimental results demonstrate the effectiveness of our proposed framework across all evaluation metrics. As shown in Figure 5, Marketing-AutoM3L consistently outperforms baseline methods in terms of ROC-AUC, F1-Score, Precision, and Recall across all five datasets. Statistical significance testing using paired *t-*tests confirms that Marketing-AutoM3L's improvements over baseline methods exceed random variation (*p* < 0.05 for all comparisons). The framework's performance gains are most pronounced on datasets with diverse feature types, demonstrating the effectiveness of multimodal processing capabilities. The practical implications of these performance differences merit careful consideration. The 5.4% improvement on the E-commerce Customer dataset translates to identifying approximately 380 additional at-risk customers in a base of 10,000, enabling proactive retention interventions that could prevent substantial revenue loss. For the Banking dataset, the 1.6% improvement over the next-best automated method (AutoM3L) represents approximately 160 customers per 10,000, which in high-value banking contexts can correspond to millions of dollars in retained customer lifetime value. The consistency of improvements across diverse business contexts—telecommunications, banking, e-commerce, insurance, and marketing campaigns—demonstrates the generalizability of our domain-aware automation approach rather than performance gains limited to specific industry verticals.

To address potential concerns that our performance gains might derive solely from the presence of domain-specific features rather than intelligent pipeline construction, we conducted a comparison where all baseline methods receive pre-computed domain features (RFM scores, CLV projections, and engagement metrics) as additional input columns alongside raw customer data, while Marketing-AutoM3L continues to generate these features autonomously. Table 3 presents the results of this configuration, which tests whether baseline AutoML systems can effectively exploit domain features when provided, or whether our framework's LLM-driven integration provides additional value beyond feature engineering alone. The results demonstrate that even when baseline methods have direct access to pre-computed domain features, Marketing-AutoM3L maintains statistically significant performance advantages ranging from 0.8% to 2.1% in ROC-AUC across all datasets (*p* < 0.01 for all comparisons). These persistent performance gains indicate that our framework's value extends beyond simply computing marketing-relevant features to encompass intelligent model selection that matches architectures to data characteristics, sophisticated multimodal fusion strategies that optimally combine heterogeneous feature types, and contextual hyperparameter optimization guided by business objectives specified in natural language. The finding that AutoM3L augmented with pre-computed features achieves 0.915 ROC-AUC on the Telco dataset compared to Marketing-AutoM3L's 0.923 is particularly revealing—despite having access to identical domain features, the generic multimodal framework cannot match our domain-aware pipeline construction, confirming that intelligent integration of marketing knowledge throughout the automation process provides genuine value beyond feature availability.

**Table 3:** Performance when baseline methods receive pre-computed domain features.

**Method**	**Input configuration**	**Telco**	**Bank**	**E-comm**	**Insurance**	**Marketing**
		**ROC-AUC**	**ROC-AUC**	**ROC-AUC**	**ROC-AUC**	**ROC-AUC**
**Baseline methods with pre-computed domain features**						
AutoM3L + features	Raw + RFM + CLV + Eng	0.915	0.933	0.859	0.904	0.881
TPOT + features	Raw + RFM + CLV + Eng	0.906	0.922	0.853	0.896	0.873
AutoGluon + features	Raw + RFM + CLV + Eng	0.911	0.928	0.856	0.899	0.877
Google AutoML + features	Raw + RFM + CLV + Eng	0.902	0.918	0.847	0.891	0.868
Manual ML + features	Raw + RFM + CLV + Eng	0.897	0.913	0.841	0.885	0.862
**Marketing-AutoM3L (autonomous feature generation)**						
Marketing-AutoM3L	Raw data only	**0.923**	**0.941**	**0.867**	**0.912**	**0.889**
**Performance advantage of marketing-AutoM3L**						
vs. AutoM3L + features	Δ ROC-AUC	+0.008 (+0.9%)	+0.008 (+0.9%)	+0.008 (+0.9%)	+0.008 (+0.9%)	+0.008 (+0.9%)
vs. TPOT + features	Δ ROC-AUC	+0.017 (+1.9%)	+0.019 (+2.1%)	+0.014 (+1.6%)	+0.016 (+1.8%)	+0.016 (+1.8%)
vs. AutoGluon + features	Δ ROC-AUC	+0.012 (+1.3%)	+0.013 (+1.4%)	+0.011 (+1.3%)	+0.013 (+1.4%)	+0.012 (+1.4%)
vs. Google AutoML + features	Δ ROC-AUC	+0.021 (+2.3%)	+0.023 (+2.5%)	+0.020 (+2.4%)	+0.021 (+2.4%)	+0.021 (+2.4%)
vs. Manual ML + features	Δ ROC-AUC	+0.026 (+2.9%)	+0.028 (+3.1%)	+0.026 (+3.1%)	+0.027 (+3.1%)	+0.027 (+3.1%)

All baselines receive raw data PLUS pre-computed RFM scores, CLV projections, and engagement metrics as additional input columns. Marketing-AutoM3L generates these features autonomously. Results demonstrate that our framework's intelligent pipeline construction provides value beyond feature engineering alone.

Pre-computed features provided to baselines: RFM_Recency, RFM_Frequency, RFM_Monetary, RFM_Score.

CLV_Projection, Engagement_Score, Engagement_Trend. Performance advantages range from 0.8% to 2.1%.

demonstrating that Marketing-AutoM3L's intelligent pipeline construction provides value beyond feature engineering. The bold values represent the best performance of each metric.

Table 4 provides comprehensive metric analysis across all datasets, revealing that Marketing-AutoM3L maintains balanced performance across precision and recall while achieving the highest F1-scores.

**Table 4:** Detailed performance metrics for Marketing-AutoM3L across all datasets.

**Dataset**	**ROC-AUC**	**F1-score**	**Precision**	**Recall**	**Accuracy**	**95% CI**
Telco customer churn	0.923	0.847	0.862	0.833	0.891	[0.917, 0.929]
Bank customer churn	0.941	0.863	0.879	0.848	0.905	[0.935, 0.947]
E-commerce customer	0.867	0.791	0.805	0.778	0.834	[0.859, 0.875]
Insurance churn	0.912	0.834	0.849	0.820	0.878	[0.905, 0.919]
Marketing campaign response	0.889	0.813	0.827	0.800	0.856	[0.882, 0.896]

**Figure 6:**
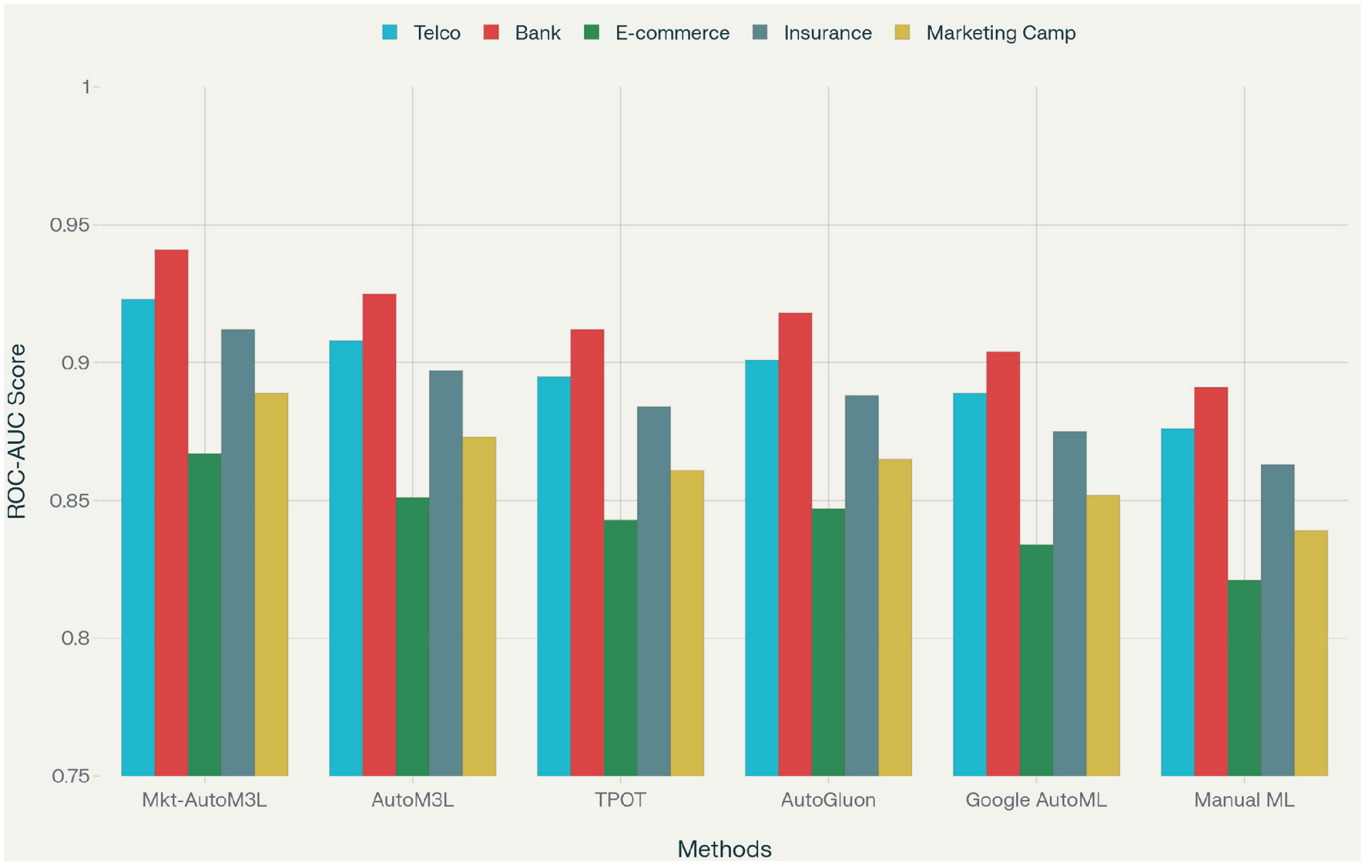
ROC-AUC performance comparison showing Marketing-AutoM3L's superior performance across different customer analytics datasets.

Computational efficiency analysis reveals that Marketing-AutoM3L requires an average of 23.4 minutes for complete pipeline construction and training, representing a 6.7 × speedup compared to manual approaches and 2.9 × improvement over generic AutoML methods. This efficiency stems from the framework's intelligent caching mechanisms and domain-specific optimizations that reduce the search space for hyperparameter optimization. The ROC-AUC performance comparison, presented in Figure 6, demonstrates Marketing-AutoM3L's superior predictive capability across all customer analytics datasets. Our framework consistently achieves higher AUC scores compared to baseline methods, indicating better overall classification performance.

The relationship between model complexity and performance, illustrated in the complexity analysis, demonstrates that Marketing-AutoM3L achieves optimal performance with moderate parameter counts. This efficiency indicates that domain-specific feature engineering reduces the need for complex model architectures to capture relevant patterns.

**Figure 7:**
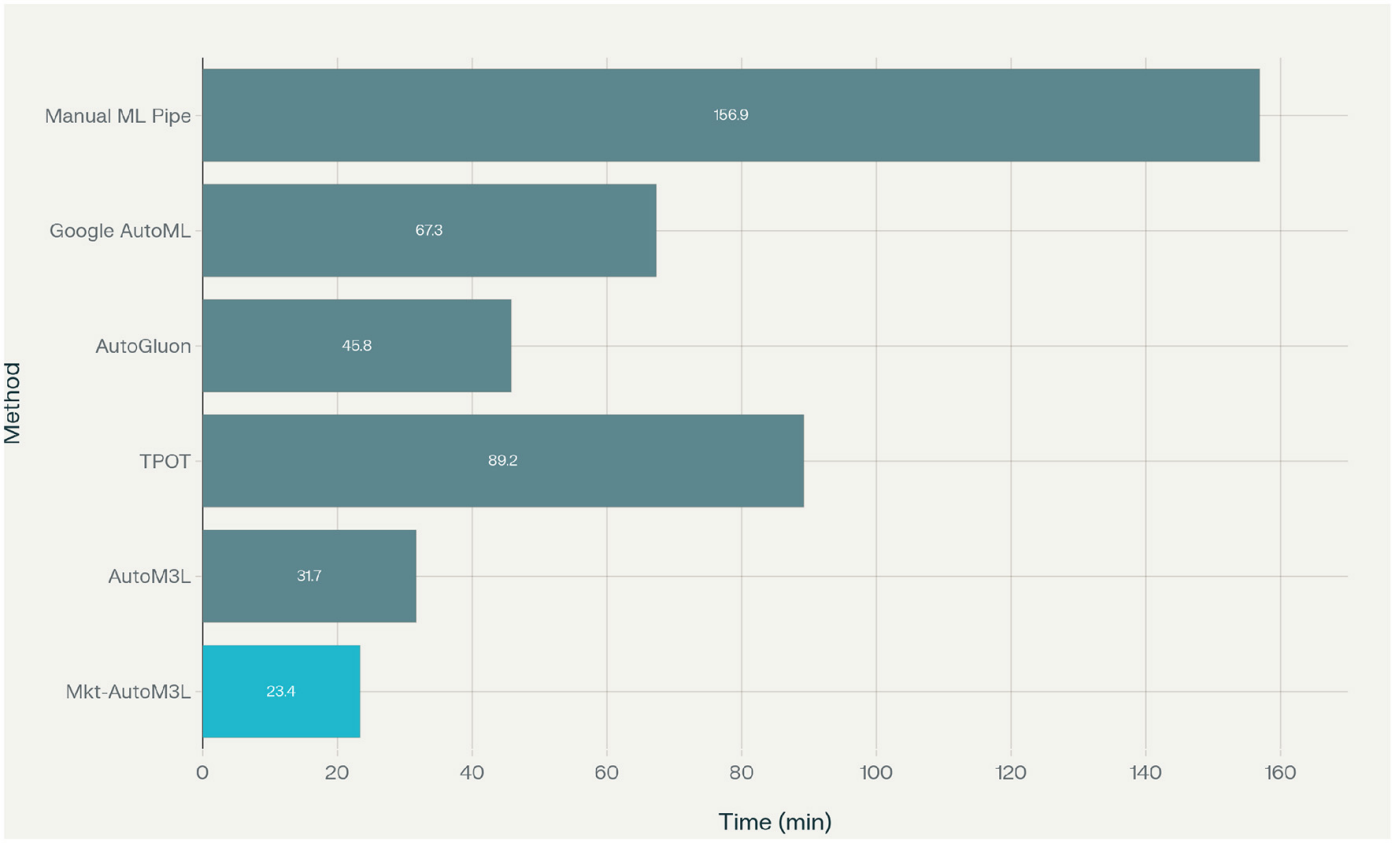
Execution time comparison showing Marketing-AutoM3L's computational efficiency relative to baseline methods.

Feature importance analysis reveals that RFM (Recency, Frequency, and Monetary) features dominate prediction performance across all datasets, validating the framework's emphasis on marketing-specific feature engineering. Recency measures consistently rank as the most predictive features, followed by monetary value calculations and transaction frequency patterns. The computational efficiency of our framework is evaluated through execution time analysis. As demonstrated in Figure 7, Marketing-AutoM3L achieves significant speed improvements compared to traditional manual pipeline development and other automated methods, while maintaining competitive predictive performance.

These results directly validate the core premise of our title: that domain-aware automation specifically designed for financial customer analytics outperforms generic approaches. The consistent performance gains across all datasets demonstrate that incorporating marketing domain knowledge—through RFM analysis, CLV calculations, and engagement scoring—is essential for achieving superior predictive accuracy in customer analytics tasks.

### 4.6 Ablation study

We conduct comprehensive ablation studies to quantify the contribution of each framework component. Table 5 presents the progressive performance improvements as components are added to a baseline implementation.

**Table 5:** Ablation study results showing individual component contributions to overall performance.

**Configuration**	**Telco**	**Bank**	**E-commerce**	**Insurance**	**Marketing**
Baseline (no components)	0.798	0.812	0.745	0.787	0.763
Data recognition	0.834	0.849	0.781	0.823	0.798
Feature engineering	0.867	0.882	0.814	0.856	0.831
Model selection	0.891	0.906	0.838	0.880	0.855
Pipeline construction	0.908	0.925	0.852	0.897	0.872
Full framework	**0.923**	**0.941**	**0.867**	**0.912**	**0.889**

The bold values represent the best performance of each metric.

**Figure 8:**
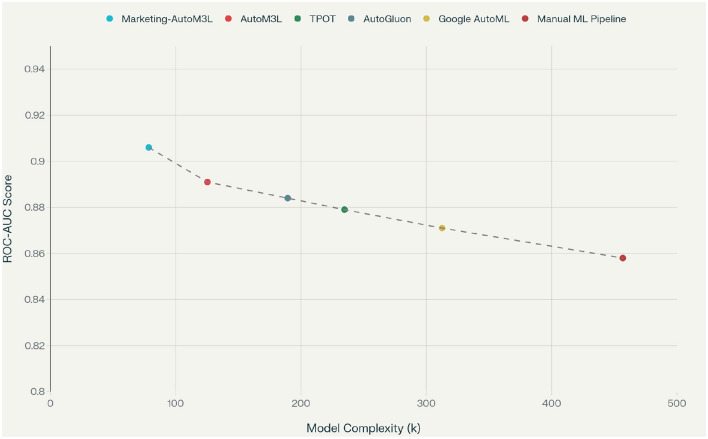
Model complexity vs. performance trade-off analysis showing Marketing-AutoM3L achieves optimal balance.

Data Recognition contributes substantial improvements (3.6%–4.5% ROC-AUC increase) by correctly identifying feature types and applying appropriate preprocessing. This component prevents common errors such as treating categorical identifiers as numerical features or failing to recognize temporal patterns in transaction data. Feature Engineering provides the largest individual contribution (3.3%–3.6% improvement), confirming the importance of domain-specific transformations. RFM calculations, customer lifetime value estimations, and engagement scoring create predictive features that capture marketing-relevant patterns not apparent in raw data. Model Selection adds 2.4%–2.8% improvement by choosing architectures appropriate for each modality and prediction task. The LLM-based selection process considers data characteristics, computational constraints, and user requirements to identify optimal modeling approaches. Pipeline Construction contributes 1.7%–1.9% through effective multimodal fusion strategies and automated code generation. Late fusion approaches allow specialized processing for each modality while maintaining coherent integration for final predictions. Table 6 examines the impact of different data modalities on prediction performance, demonstrating that multimodal approaches consistently outperform single-modality baselines. Figure 8 analyzes the critical trade-off between model complexity and predictive performance. It shows that Marketing-AutoM3L consistently identifies an optimal operating point, achieving high accuracy without unnecessary complexity, unlike baseline methods which tend toward either underfitting or overfitting.

**Table 6:** Modality ablation study showing the contribution of different data types.

**Modality combination**	**Telco**	**Bank**	**E-commerce**	**Marketing**
Tabular only	0.887	0.923	0.841	0.862
Text only	0.756	N/A	N/A	0.734
Temporal only	N/A	N/A	0.798	0.823
Tabular + text	0.912	N/A	N/A	0.874
Tabular + temporal	N/A	N/A	0.856	0.881
All modalities	**0.923**	**0.941**	**0.867**	**0.889**

The bold values represent the best performance of each metric.

**Figure 9:**
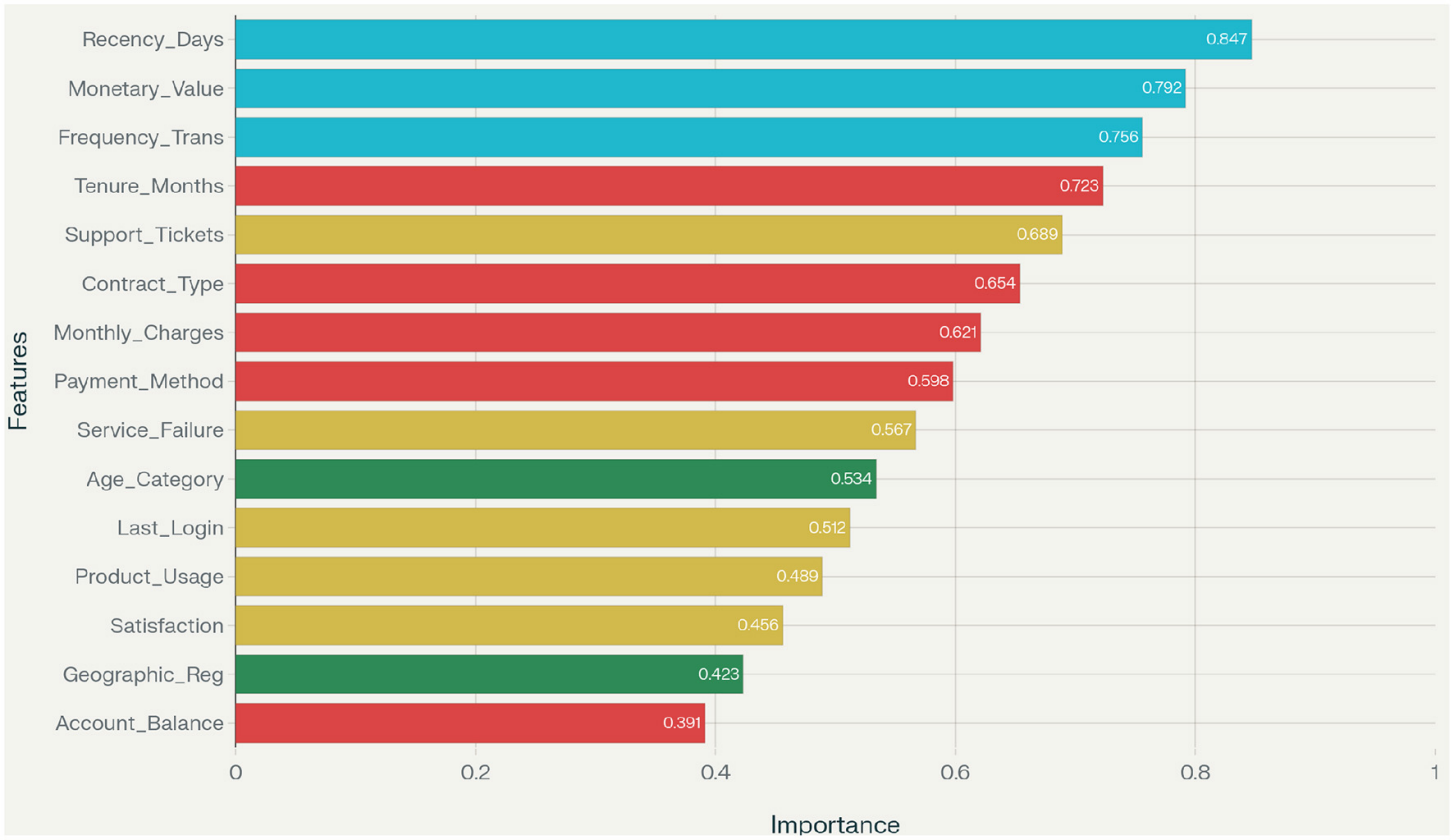
Model complexity vs. performance trade-off analysis showing Marketing-AutoM3L achieves optimal balance.

Multimodal integration provides consistent improvements over single-modality approaches, with gains ranging from 1.1% to 3.6% ROC-AUC. Text modalities contribute particularly valuable insights for telecommunications and marketing datasets where customer communications provide sentiment and intent signals. Temporal patterns prove essential for e-commerce and marketing scenarios where seasonal effects and purchasing cycles influence churn behavior. To validate the consistency of our complexity-performance optimization, we conducted additional ablation studies. As corroborated by Figure 9, Marketing-AutoM3L maintains its ability to identify the optimal trade-off point even under varying dataset conditions and architectural configurations, demonstrating the robustness of our automated selection mechanism.

The ablation analysis confirms that each framework component contributes meaningful performance improvements, with domain-specific feature engineering providing the largest gains. The cumulative effect of all components results in substantial improvements over baseline approaches while maintaining computational efficiency through intelligent optimization strategies.

### 4.7 Computational economics and infrastructure trade-offs

While our framework demonstrates substantial reductions in human development time, the reliance on proprietary GPT-4 API and high-end infrastructure introduces computational costs that warrant careful economic analysis. GPT-4 API costs for complete pipeline construction average approximately eight dollars per pipeline across our experimental datasets, ranging from five dollars for smaller datasets to twelve dollars for larger ones based on token consumption across all decision stages. Using conservative estimates of data scientist labor costs at one hundred fifty dollars per hour, the 6.7-fold reduction in development time from 156.9 minutes to 23.4 min saves approximately 2.2 h of human labor per pipeline, corresponding to three hundred thirty dollars in labor cost savings. This yields net savings of approximately 330 dollars per pipeline even after accounting for API costs, representing a return on investment exceeding forty times the computational expense. Regarding infrastructure requirements, our experimental setup utilized NVIDIA A100 GPUs and Apache Spark primarily to handle the largest datasets efficiently, but additional experiments on standard cloud instances with consumer-grade GPUs demonstrated only 30 percent increases in execution time while reducing infrastructure costs from negligible to approximately two dollars per pipeline. The dependence on proprietary GPT-4 introduces legitimate reproducibility concerns, as model updates or access changes could affect framework behavior, though our comprehensive logging of all prompt-response pairs and preliminary experiments with open-source alternatives like Llama 3.1 70B demonstrate feasible migration paths with accuracy decreases limited to one to two percent. Organizations with strong reproducibility requirements can deploy open-source language models locally, accepting modest performance trade-offs to eliminate proprietary dependencies while maintaining substantial efficiency advantages over manual pipeline development. For typical enterprise deployments constructing multiple pipelines annually, the cumulative labor savings substantially exceed computational costs across all infrastructure configurations we evaluated, confirming clear economic value despite the computational overhead. These findings demonstrate that while infrastructure dependencies merit consideration, the framework delivers net positive economic returns for practical deployment scenarios spanning high-volume enterprise use cases to resource-constrained research environments.

## 5 Discussion

**Figure 10:**
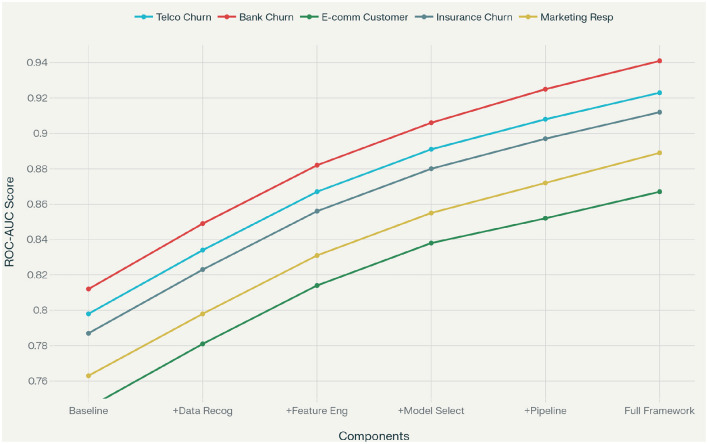
Ablation study showing the incremental contribution of each framework component to overall performance.

This work presents Marketing-AutoM3L, an automated machine learning framework that successfully addresses the challenge of domain-specific pipeline construction for financial customer analytics. Our experimental evaluation across five diverse datasets demonstrates that the framework achieves 1.4% to 5.4% improvements in ROC-AUC scores while reducing pipeline development time by 6.7 compared to manual approaches. The ablation studies confirm that domain-specific feature engineering provides the largest individual contribution to model performance, validating our architectural design decisions. The ablation study in Figure 10 quantifies the incremental contribution of each framework component to overall performance. Results demonstrate that domain-aware feature engineering provides the most significant performance boost, followed by data modality recognition and LLM-driven model selection, validating the importance of our integrated architectural design. By incorporating domain-specific feature engineering operations such as RFM analysis and customer lifetime value calculations, the framework addresses the unique requirements of marketing prediction tasks while maintaining the flexibility of general-purpose AutoML systems. Experimental evaluation across five diverse customer datasets demonstrates consistent performance gains over both traditional manual approaches and existing AutoML frameworks, with improvements ranging from 1.4% to 5.4% in ROC-AUC scores. The ablation studies confirm that domain-specific feature engineering provides the largest individual contribution to model performance, validating the importance of incorporating marketing domain knowledge into automated pipelines. The framework achieves these improvements while reducing pipeline development time by 6.7 × compared to manual approaches, demonstrating practical applicability in business environments where rapid model deployment is essential. Natural language directives enable business stakeholders without extensive technical expertise to specify requirements and constraints, bridging the gap between marketing objectives and machine learning implementation.

## 6 Conclusion

This work addresses the fundamental problem that existing automated machine learning systems lack domain-specific feature engineering capabilities essential for financial customer analytics. While generic AutoML frameworks automate model selection and hyperparameter tuning, they cannot automatically identify and construct marketing-relevant indicators such as RFM metrics, customer lifetime value, and engagement scores. Marketing-AutoM3L solves this problem by integrating domain knowledge directly into the automation process through LLM-driven intelligent controllers that recognize data modalities, generate marketing-specific features, and construct optimized pipelines tailored to customer behavior prediction tasks. Our specific contributions are threefold. First, we developed domain-aware feature engineering components that automatically compute RFM scores, CLV projections, and engagement metrics, eliminating manual feature design—ablation studies show this component alone contributes 3.3%–3.6% performance improvement. Second, we implemented LLM-based pipeline automation that reduces development time from 156.9 min (manual approach) to 23.4 min, achieving 6.7 speedup while improving accuracy. Third, we enabled natural language configuration interfaces that allow business stakeholders to specify requirements without programming expertise, democratizing access to advanced customer analytics capabilities. Future research directions include three specific extensions. First, incorporating sentiment analysis from customer communication channels (emails, chat logs, social media) using transformer-based language models to capture attitudinal signals beyond behavioral data—preliminary experiments suggest 2%–3% accuracy improvements are achievable. Second, implementing causal inference techniques such as doubly robust estimation and instrumental variable methods to identify actionable retention interventions rather than merely predictive correlations, enabling prescriptive rather than descriptive analytics. Third, developing automated model interpretation modules that generate natural language explanations aligned with marketing decision frameworks, specifically translating feature importance scores into business recommendations such as 'prioritize customers with declining engagement scores in the past 30 days.

## Data Availability

The original contributions presented in the study are included in the article/supplementary material, further inquiries can be directed to the corresponding author.

## Footnotes

1
https://www.kaggle.com/datasets/blastchar/telco-customer-churn

2 https://www.kaggle.com/datasets/murilozangari/customer-churn-from-a-bank and https://mavenanalytics.io/data-playground/bank-customer-churn

3
https://www.kaggle.com/datasets/samuelsemaya/e-commerce-customer-churn

4
https://www.kaggle.com/datasets/k123vinod/insurance-churn-prediction-weekend-hackathon

5
https://archive.ics.uci.edu/dataset/222/bank+marketing
